# Carrier Mapping in Sub‐2nm Node Nanosheet Transistors with Scanning Spreading Resistance Microscopy

**DOI:** 10.1002/smtd.202502279

**Published:** 2026-02-10

**Authors:** Andrea Pondini, Pierre Eyben, Lennaert Wouters, Albert Minj, Thomas Hantschel, Philippe Matagne, Jérôme Mitard, Anne Verhulst

**Affiliations:** ^1^ IMEC vzw Leuven Belgium; ^2^ Department of Electrical Engineering (ESAT) KU Leuven Leuven Belgium

**Keywords:** carrier mapping, Complementary‐FETs (CFETs), gate‐all‐around (GAA), junction profiling, Nanosheet‐FETs (NSFETs), scanning spreading resistance microscopy, sub‐2 nm semiconductor nodes

## Abstract

As the semiconductor industry transitions to gate‐all‐around architectures such as Nanosheet‐FETs (NSFETs) for the 2nm node and beyond, controlling parasitic resistance through precise junction engineering is fundamental. This requires characterization methods capable of mapping active carriers with nanometer‐scale resolution. This work demonstrates a significant advancement in scanning spreading resistance microscopy (SSRM) that enables, for the first time, carrier mapping within 5.5 nm thick nanosheet channels. This was achieved through a systematic optimization of sample preparation to achieve sub‐nanometer topography, the use of ultra‐sharp diamond probes, and the implementation of a linear current amplifier to eliminate artifacts from slow logarithmic amplifiers. SSRM measurements of NSFETs with and without a 950°C rapid thermal anneal reveal a clear increase in phosphorus diffusion due to the higher thermal budget, with carrier profiles in excellent agreement with Kinetic Monte Carlo process simulations. This demonstrates how SSRM is a valuable characterization technique for providing direct feedback on junction formation in advanced gate‐all‐around devices.

## Graphical abstract

Within this work, advancements in scanning spreading resistance microscopy (SSRM) allow charge carrier mapping within 5.5 nm‐thick nanosheet channels. Devices subjected to rapid thermal annealing at 950°C show ∼ 5 nm enhanced phosphorus diffusion, with profiles in close agreement with semi‐atomistic process simulations. This establishes SSRM as a unique technique that provides direct insight into junction formation in advanced gate‐all‐around architectures.

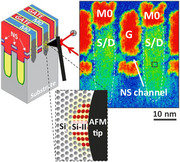

## 1 Introduction

For decades, transistor scaling enabled increasing the number of devices on a chip and thus complying with the growing compute and memory requirements of advancing technologies. For the 2nm logic node and beyond, the industry is moving from the FinFET architecture to gate‐all‐around (GAA) transistor architectures such as Nanosheet‐FETs (NSFETs) [1, 2, 3, 4] and Complementary‐FETs (CFETs) [5, 6, 7]. In these 3D structures, the transistor channels are vertically stacked and fully embedded in gate metal. However, due to the reduced device dimensions, parasitic resistance components, such as source/drain (S/D) resistances and extension resistances, have an increasingly hampering effect on device performance [8, 9]. Doping engineering is therefore key and consists of minimizing the resistance of the regions which are not gate controlled by maximizing the active dopants and carefully controlling the dopant diffusion towards the device channel without inducing short‐channel effects [10, 11, 12]. In scaled NSFETs, even 1–2 nm errors in the junction placement can degrade the drive/leakage current ratio and significantly affect circuit‐level performance [13]. Therefore, characterization techniques that allow to map charge carriers and observe their placement in the channels of nanosheet‐based devices are of critical importance for performance optimization [14].

Characterization techniques should comply with stringent requirements to be considered as viable options for device junction characterization [10, 14, 15]. A key constraint is the spatial resolution required to image the nanosheet channels. In addition, the sensitivity and dynamic range must be sufficient to measure dopant concentration variations spanning over several orders of magnitude. Lastly, the techniques should provide reproducible results and be applicable to relevant test‐structures and devices at the end of the manufacturing process. One of the most widespread and well‐established techniques in the field of chemical mapping with atomic spatial resolution is transmission electron microscopy (TEM) based electron energy loss spectroscopy (EELS) [16, 17]. Another powerful technique is atom probe tomography (APT), which provides 3D spatial reconstructions of the positions of individual atoms within the sample. Although this technique has been demonstrated for dopant mapping in FinFETs and silicon nanowires [18, 19, 20], the atom trajectory reconstruction is currently a limitation of the technique, especially when atoms with vastly different evaporation rates are adjacent to each other [19, 20, 21]. This is particularly true in devices for sub‐2nm logic nodes, where silicon nanosheet channels are stacked and fully embedded in gates with multiple metallic and dielectric layers [22] Furthermore, since only one (or part of a) device can be analyzed at once during an APT measurement, the resulting information is subject to stochastic effects from processing (geometric inhomogeneities, random dopant fluctuations, etc.) and ion detection efficiency. Both APT and EELS can provide the local spatial chemical concentration of dopants but no information on electrically active charge carriers, which can significantly differ due to dopant deactivation and carrier trapping [11, 23, 24, 25, 26].

One technique used to extrapolate information about carriers is TEM‐based electron holography, which measures the phase difference between electron beams passing through doped regions of the semiconductor devices [27, 28]. Typically, the measured signal is strongly affected by the materials and morphology of the devices under investigation, making it challenging to characterize the channel of nano‐devices at the end of the manufacturing process and to correlate the measured potential signal with carrier concentrations. On the other hand, atomic force microscopy (AFM)‐based techniques such as scanning spreading resistance microscopy (SSRM) [15, 28, 29], scanning capacitance microscopy (SCM) [28, 30] or scanning nonlinear dielectric microscopy (SNDM) [31], scanning microwave impedance microscopy (SMIM) [32], and scattering scanning near field optical microscopy (s‐SNOM) [33, 34, 35] are different approaches for mapping carriers in semiconductor materials. In particular, SSRM measures the spreading resistance underneath the probe, leveraging the phase transformation of silicon from semiconducting to metallic when under high pressures, which lowers the contact resistance and improves spatial resolution [29, 36]. In practical high‐resolution SSRM operation, the applied mechanical load is carefully controlled to remain in an imaging regime that preserves both the device cross‑section and the probe integrity. By applying a voltage difference between the probe and the sample and collecting the resulting current using a logarithmic amplifier, SSRM maps the spreading resistance over several orders of magnitude, which can be quantitatively linked to active carrier concentrations through the use of suitable calibration samples [37]. Even though such quantitative calibration cannot be applied to scaled devices due to their confined geometry and devicespecific current paths, the SSRM signal remains highly powerful for comparing different samples or process conditions under identical measurement settings. Owing to its combination of nanometer‐scale spatial resolution and large dynamic range, SSRM has evolved into a powerful technique for carrier profiling in planar and FinFET transistor architectures [29]. High‐resolution SSRM measurements have successfully imaged advanced FinFET devices [38, 39] and GAA transistors with relaxed dimensions based on nanowires with 50 nm length and 10 nm thickness [40]. However, the spatial resolution of SSRM has not kept pace with the continued scaling of state‐of‐the‐art transistors, where shrinking dimensions and the increasing presence of interfaces and heterogeneous materials have limited the applicability of the technique in the most advanced nodes.

In this work, we present how recent SSRM developments, including optimized test structures, tailored sample preparation, dedicated AFM probes, and an alternative current amplifier, now unlock carrier mapping in sub‐2nm node NSFETs. This advancement enables us to capture the impact of varying thermal budgets on dopant diffusion inside state‐of‐the‐art GAA devices. Samples with different processing conditions are analyzed and compared to advanced semi‐atomistic process simulations, demonstrating SSRM's capability to resolve carrier distributions in GAA transistors with channels twice as thin and four times shorter than measured before. These improvements establish SSRM as a powerful characterization method to evaluate process alternatives for junction engineering in advanced semiconductor nodes.

## 2 Methods

The devices investigated in this work are single nanosheet nMOS NSFET devices described in detail elsewhere [11]. The NS channel dimensions satisfy A10 logic node specifications [22]: gate pitch of 45 nm, gate length of 12 nm, nanosheet width of 18 nm and nanosheet thickness of 5.5 nm. The devices S/D regions consist of epitaxially grown phosphorus‐doped silicon (Si:P), with sample A and sample B denoting samples without and with an additional rapid thermal anneal (RTA) step at 950°C for 1.5 s following S/D epitaxy, respectively.

**FIGURE 1:**
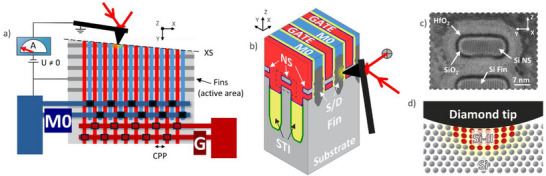
a) Schematic of SSRM test‐structure and measurement. b) 3D view of the SSRM probe scanning on the exposed NSFET cross‐section, on the surface parallel to the channel direction. c) Annular bright field scanning tunneling electron microscopy (ABF‐STEM) image perpendicular to the channel direction, corresponding to the dashed rectangle in b). d) Schematic of the pressure induced metallic silicon pocket below the AFM diamond tip.

A dedicated SSRM test structure is designed, which is shown and described in Figure S1, along with the procedure employed to process a back‐contact for SSRM measurements. During SSRM measurements, the AFM probe is grounded, and the bias is applied to the sample via the back‐contact (Figure 1a) while the probe scans perpendicular to the gate direction (Figure 1b). AFM measurements are carried out on a Bruker Dimension Icon‐PT AFM system in a glovebox with Ar environment (pressure > 1 bar and residual H_2_O and O_2_ concentrations lower than 0.1 ppm). Before SSRM measurements, the samples are kept in a vacuum load lock in high vacuum for several hours to remove any adsorbed water layers. This is particularly important since the water layer that forms on the sample surface is believed to inhibit the pressure‐induced transition from semiconducting Si to metallic Si‐II (also called β‐Si or β‐Sn phase) at the core of SSRM [41]. In addition, the presence of water might affect the required probe force for establishing a stable contact, either due to the higher force needed to punch though the water layer [42], or because of the associated capillary forces [43]. Measurements are carried out with two types of commercially available boron‐doped diamond (BDD) probes: cone‐shaped Adama FM LC with nominal spring constant, *k*, of 8 N/m and tip radius, *r_tip_*, of 20 ± 10 nm, and apex sharp Adama AD‐2.8‐AS with *k* = 2.8 N/m and *r_tip_* = 10 ± 5 nm. These two kinds of probes will be referred to as *cone* and *sharp* probes further along in the text. The PF‐TUNA and SSRM application modules from the AFM tool manufacturer were used to collect the current flowing through the probe, which will be referred to as *linear* and *logarithmic* current amplifiers, respectively.

## 3 Results and Discussion

### 3.1 Cross‐Section Preparation

Proper cross‐section preparation is essential to minimize topography effects and improve SSRM spatial resolution. The former is required to avoid the topography artifacts are induced in the SSRM signal, and to avoid the need for additional flattening through AFM scalpelling and material removal, which can damage both the AFM probe and the sample surface [44, 45]. Three typical ways of preparing smooth integrated circuit cross‐sections are micro‐cleaving, mechanical polishing and FIB milling [46, 47]. In this work, FIB cross‐section preparation is not considered due to the induced beam damage on the sample cross‐section: it has been shown that, even at low beam energies of 5 keV, at least the upmost 5 nm of silicon gets amorphized, and that the spreading resistance measured on the layers underneath can be several orders of magnitude different from what is measured on pristine layers, skewing the apparent carrier concentrations measured [48]. For this reason, we here compare the topography of cross‐sections obtained with micro‐cleaving and polishing methods. To do so, we employ PeakForce tapping AFM [49], performed in air with cone probes.

**FIGURE 2:**
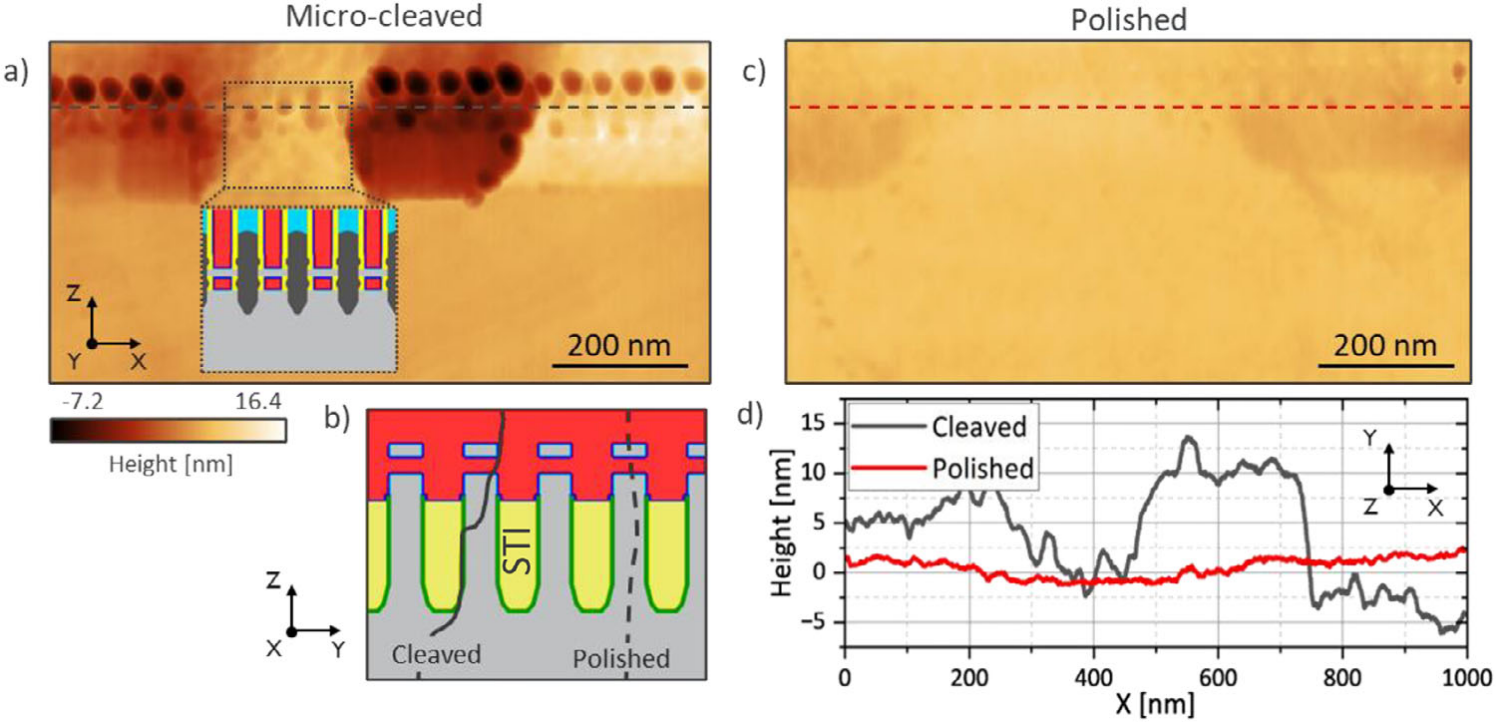
AFM topography images of the cross‐section of an NSFET SSRM test‐structure prepared by (a) micro‐cleaving and (c) polishing methods. The level of the substrate was set at 0 nm in the height scale. b) Examples of possible cleaving (solid line) and polishing (dashed line) surfaces though the devices active area. d) Height profiles extracted along the dashed lines in Figure a) and c).

Micro‐cleaving is a fast and easy way to obtain cross‐sections of silicon layers with low surface roughness [46], as also confirmed in Figure 2a where the root mean square (RMS) roughness in the Si substrate is < 4 Å (over a 3×3 µm area). One difficulty arises when multiple heterogeneous materials are closely placed next to each other on the resulting cross‐section, as the difference in ductility and hardness at the nanoscale is reflected in inhomogeneous cleaving behavior. Figure 2a shows that this results in surfaces with peak‐to‐peak roughness exceeding 10 nm in height in the devices active area. This is because while in the substrate the cleaving follows a well‐defined surface, in the active area, due to the heterogeneity of the materials, the cleaving surface propagates very irregularly, resulting in topography features. A sketch of this behavior is illustrated with a solid line in Figure 2b. One consequence is that the cleaving surface often lands outside of the region of interest, for example in the shallow trench isolation (STI) that separates devices.

By contrast, cross‐section polishing can be performed while mounting the sample at a small angle (<1 deg) so that the polishing plane intersects the active area of the devices at different positions along the beveled surface, making it convenient to find a suitable position for SSRM measurements (see Figure 1a). Prior to mechanical polishing, a glass dummy is glued onto the coupon to minimize cross‐section rounding effects [47]. The polishing is performed using lubricant and diamond polishing papers of different grain sizes, starting from 30 µm grains down to 0.1 µm. A final polishing step with aluminum oxide paper (grain size of 0.01 um) allows to reach a surface RMS roughness < 3 Å (measured over a 3×3 µm area), although some deeper polish lines (∼1 nm depth) can be observed on the sample surface. Figure 2c shows that, contrary to micro‐cleaving, topography features in the active area of polished devices are strongly reduced. By comparing line profiles in the devices active area (Figure 2d) one can see that polishing results in topography features with a height of around 1 nm, significantly smaller in comparison to micro‐cleaving. For this reason, polishing is chosen as primary method for cross‐section preparation in our study. One drawback of this approach is the introduction of surface states on the polished silicon surface [50, 51]. Although the associated charge might impact the carrier profiling in layers lowly doped with acceptors, this is not relevant for the measurement of the highly doped regions in the devices of this study.

### 3.2 AFM Probes and SSRM Radius

**FIGURE 3:**
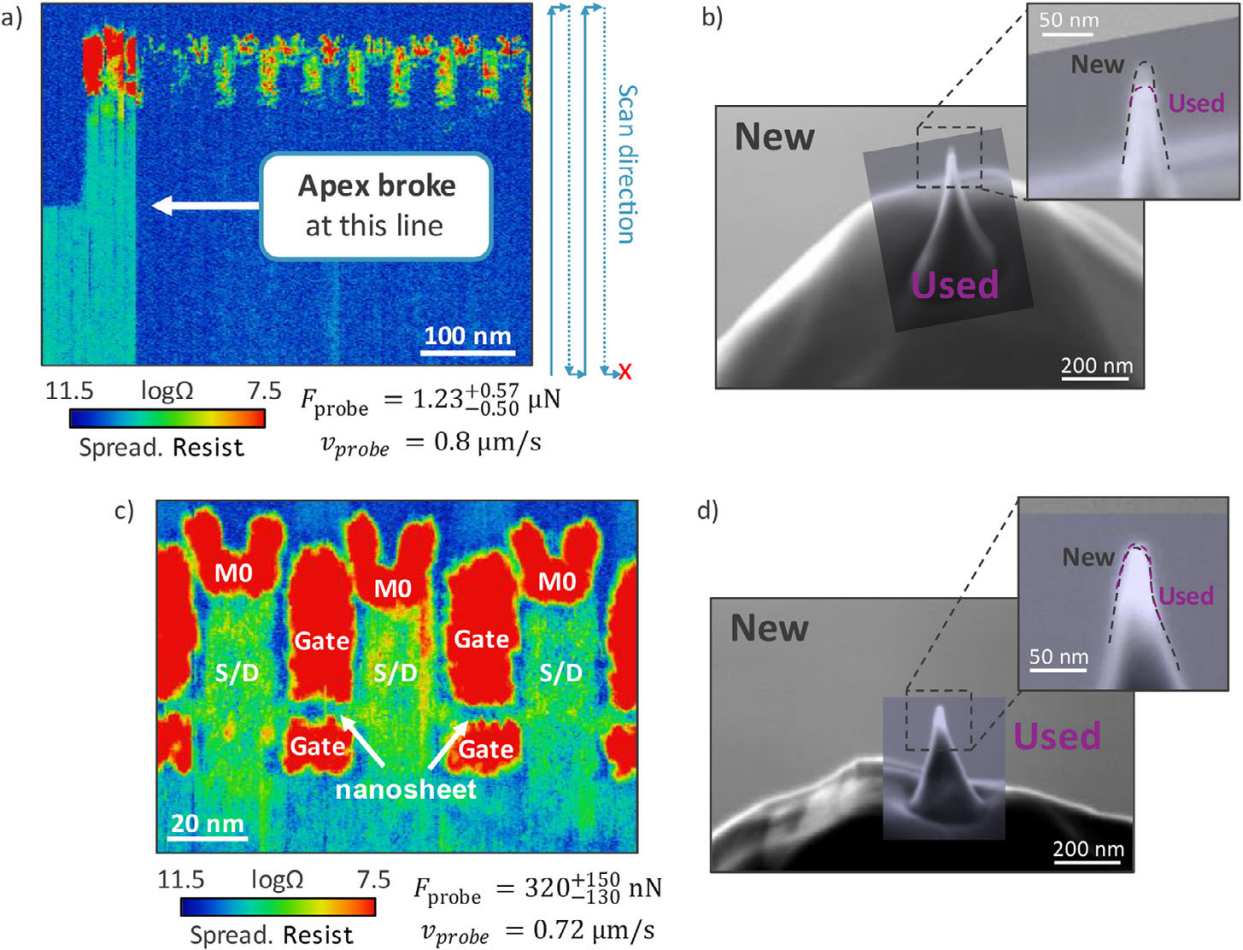
a,b) SSRM image of NSFET transistors at high force and SEM inspection of the AFM probe employed during the measurement. The SSRM contrast and spatial resolution suddenly change after some scan‐lines, suggesting that AFM probe suffered a modification. By overlaying SEM images of the probe before and after it was used, it's visible how the sharp apex broke due to the high forces during SSRM scanning. c, d) SSRM image at lower force and corresponding SEM inspection of the probe employed, which in this case is intact and does not show visible modifications.

The optimal surface preparation achieved with polishing methods allows direct access to the area of interest. This enables the use of sharper diamond probes, which would otherwise risk being damaged under the higher pressures (on the order of tens of GPa) required for cutting through Si material [44, 45] We show in Figure 3 that high resolution images can be obtained with forces that are one order of magnitude lower compared to the typical force ranges used for traditional cone (or pyramidal) full diamond or diamond‐coated probes [46, 52], greatly improving resolution.

Before achieving successful SSRM measurements, the AFM deflection setpoint is gradually increased until the force applied by the probe is sufficient to observe stable contrast in the acquired spreading resistance signal. The threshold force is specific to each probe due to the specific morphology of the apex, resulting in different applied pressures. In Figure 3a, stable contrast was briefly observed by applying a typical force, *F_probe_*, of around 1.2 µN. Shortly after, both the spatial resolution and the spreading resistance signal suddenly degraded during the data acquisition, suggesting a sudden apex modification. In fact, if a portion of the apex breaks off and the contact radius increases, a lower pressure is induced in the silicon, which is insufficient to induce the phase change to metallic silicon. This is confirmed by scanning electron microscopy (SEM) inspections: Figure 3b shows an overlay of SEM images of the sharp diamond probe obtained before it was used for measurements, and after the sudden change in spreading resistance and resolution was observed. By comparing the two images it's possible to observe that, while a sharp protrusion is maintained, a clear change in probe apex occurred, which is now 20 to 25 nm shorter compared to its pristine condition. The SSRM image suggests that this can be attributed to a sudden breakage of the diamond protrusion. Following this event, the probe was not suitable for obtaining the resolution needed to measure inside the nanosheet channel of the scaled NSFET devices under investigation but was still functional.

Repeating the SSRM measurement with a new sharp probe, but now with a significantly lower force of ∼320 nN, results in the SSRM map of Figure 3c, which clearly distinguishes all the parts of the NSFETs, including the nanosheet channels. Note that the applied force in this case is one order of magnitude lower compared to the typical ∼µN force ranges used when using traditional cone (or pyramidal) full diamond or diamond‐coated probes [46, 52]. Figure 3d displays SEM images of the sharp probe employed to capture Figure 3c. No clear change can be observed in the probe apex from before and after the SSRM measurement was performed. This observation illustrates the delicate balance between the applied force, probe morphology, and SSRM performance. This also hints at the fragile equilibrium between probe sharpness and mechanical robustness, suggesting that the use of even sharper probes may be futile due to their incline to breakage when subjected to excessive stress. Instead, maintaining apex integrity and controlling the applied force are key to achieving reliable and high‐resolution SSRM imaging in scaled devices. On the other hand, Figure 3b also shows that sharp diamond probes can retain a usable apex even in the case of partial breakage, which allows them to continue functioning for SSRM measurements, albeit with reduced resolution. This endurance may explain their application in other SSRM studies, with higher forces and lower resolutions.

A simple estimation of the tip‐sample contact radius can be achieved using the Derjaguin‐Muller‐Toporov (DMT) model [53]. If we consider the probe apex as a sphere with radius *r_tip_* indenting the sample planar surface with a force *F*, the contact radius between the two (*r_c_*) is equal to:

(1) $$r_{c}=\sqrt[{3}]{\frac{r_{tip}}{K}\left(F+2\pi r_{tip}W\right)}\;,$$

with

(2) $$\frac{1}{K}=\frac{3}{4}\;\left(\frac{1-\nu_{1}^{2}}{E_{1}}+\frac{1-\nu_{2}^{2}}{E_{2}}\right)$$

Here *ν_1_*, *ν_2,_* and *E_1_*, *E_2_* are respectively the Poisson's ratios and Young's moduli of diamond and silicon, and *W* the work of adhesion. The values used for *ν* and *E* are reported in Table 1, and the value used for work of adhesion is *W* = 0.66 J/m2 [54]. Using the AFM probe manufacturer specifications, we can estimate that for a force of 300 nN the contact radius is *r_c_* ≈ 2.5 ± 0.5 nm, with probe indentation depth into the silicon *δ* = *r_c_* ^2^/*r_tip_* < 1 nm (see Table 2). The DMT model suggests that the adhesion contribution to the contact radius ranges from ∼2% to ∼6%, corresponding to the lower and upper bounds of the specified probe radius. Although the Si‐I to Si‐II phase transition typically occurs between 11 and 14 GPa in hydrostatic conditions [55, 56, 57, 58], shear stresses can significantly affect this threshold [57, 59, 60, 61] The peak static pressure (at the center of the contact) can be estimated by the DMT model as *P* = 3*F*/2π*r_c_*, which exceeds this threshold for the force and probe radius mentioned above (see Table 2).

**TABLE 1:** Parameters used for the modelling of the probe‐sample contact.

	Silicon ⟨110⟩	Boron‐doped diamond
Poisson's ratio (ν)	0.28 [56]	0.072 [57]
Young's modulus (*E*)	169 GPa [56]	1108 GPa [57]

**TABLE 2:** Estimation of contact radius, indentation and peak static pressure using the DMT model when sharp diamond probes indent silicon with a force of 300 nN.

	*r_c_*	Adhesion contribution	δ	Peak pressure
5 nm^b)^	2.0 nm	2.3%	0.78 nm	37 GPa
10 nm^a)^	2.5 nm	4.4%	0.64 nm	22 GPa
15 nm^c)^	3.0 nm	6.5%	0.58 nm	16 GPa

^a)^ Expected value.

^b)^ Lower and.

^c)^ Upper limit of the manufacturer specifications.

Note that the value used for adhesion does not consider possible native oxides or surface contaminations that might affect adhesion forces, neither does the model account for plastic deformation or shear stresses due to AFM scanning. It is therefore an approximation, yet it provides a rough estimate of the contact area between the diamond probe and silicon during SSRM measurements. It is worth noting that in conductive AFM techniques, the effective area through which the current flows is often different compared to the contact area [43] In SSRM, the effective radius of the electrical interaction is given by the pressure‐induced metallic silicon pocket, which is small compared to the contact area at small forces as confirmed by molecular dynamics simulations [36, 61, 62]. For example, it was shown [62] that for a probe radius of 10 nm (consistent with the probes used in this study) and an indentation depth of 2 nm, the metallic silicon atoms mostly concentrate within a region of radius *r_Si‐II_* ∼ 2.7 nm, which is 40% smaller than the contact radius (*r_c_*) of 4.5 nm. The ratio *r_Si‐II_*/*r_c_* is expected to be even smaller in our experiments, due to the sharp increase of metallic atoms at small indentation depths [36], suggesting a value of *r_Si‐II_* in the range of 1 nm, which is a range in full alignment with our experimental observations in Figure 3c, where the 5.5 nm thick transistors’ channels are successfully imaged.

### 3.3 Limits of SSRM Logarithmic Current Amplifier

**FIGURE 4:**
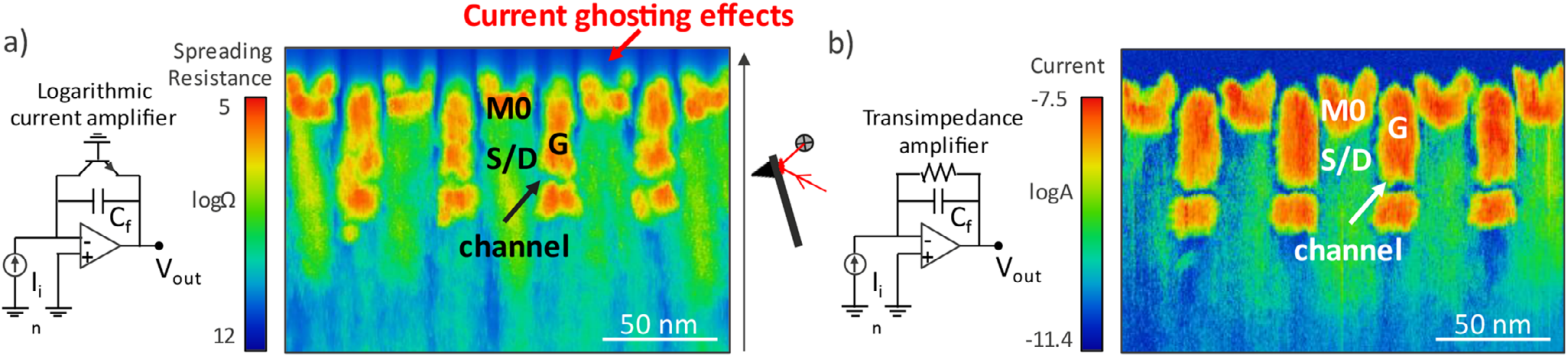
a) Simplified schematic of a logarithmic current amplifier and associated SSRM image. Current ghosting effects are seen when scanning from metallic into oxide regions due to the slow response time of the amplifier at low currents. b) Simplified schematic of a transimpedance current amplifier and associated image, with no current ghosting effects due to the faster response time.

Although the SSRM technique has relied on the use of a logarithmic amplifier since its invention three decades ago [29], the carrier distribution and geometry of advanced devices may necessitate a revision of this approach. Figure 4 compares two SSRM acquisitions on the same area of test‐structures, acquired using a logarithmic (Figure 4a) and a linear current amplifier with current sensitivity set to 2 nA/V (Figure 4b). Here the AFM scanning direction is from bottom to top. By comparing measurement acquisitions, we can observe that in Figure 4a the spreading resistance signal is blurred and convoluted with current ghosting effects. This is particularly clear in the oxide region above the device's metallic S/D contacts and gates, where instead of measuring high values of resistance (insulating layers) we see trailing current effects due to the amplifier's slow response time. These ghosting effects stem from the design of the logarithmic amplifier, which core functioning relies on the substitution, compared to a linear transimpedance amplifier (TIA), of the feedback resistor with an active component such as a BJT transistor (or a diode) [63, 64]. A small capacitor *C_f_* is commonly added to stabilize the op‐amp loop [68]. We can estimate the effective time constant for the op‐amp and feedback network as:

(3) $$\tau =r_{e}\;C_{f}=\frac{dV_{BE}}{dI_{C}}\;C_{f}\approx \left(\frac{V_{t}}{I_{in}}\right)C_{f},$$

where *r_e_* is the dynamic resistance at the BJT emitter, *V_t_* ≈ 26 mV is the thermal voltage and *I_in_* the input current into the amplifier. The formula shows that this response time can become huge when measuring small currents, reducing the bandwidth of the amplifier. If we consider a typical value of *C_f_* = 50 pF [63] and a scanning speed of 1 µm/s, when measuring currents of 10 nA (for example over a S/D region) the response time is ∼ 0.1 ms, corresponding to a negligible blur of 0.1 nm. However, for a low current of 25 pA, typical of lowly doped/near‐intrinsic region, the response time increases to 50 ms, resulting in a 50 nm artifact trail, completely obscuring the nanoscale features of interest. This distance could be partially reduced by reducing the scanning speed, although this often results in AFM thermal drift, inconsistency in SSRM signal, and significantly longer measurement times. At the same time, lowering the value of *C_f_* can result in amplifier instabilities and oscillations [63]. The slow response time when measuring small currents is thus an intrinsic limitation of logarithmic amplifiers. This obstacle is now more relevant for the measurement of advanced transistors compared to previous technologies, as the required high spatial resolution results in small probe‐sample contact radii and low measured currents. In addition, the heterogeneity in materials in the active area due to the gate‐all‐around nature of nanosheet‐based transistors requires that the amplifier is fast enough to respond to large variations in input current. For example, when scanning from the substrate to the top of the devices, as was the case in Figure 4, in the channel region the AFM probe will first encounter the bottom part of the gate before moving onto the transistor channel. The two are only separated by a ∼ 2 nm dielectric layer (see Figure 1c), which is insignificant when compared to the distances required for the logarithmic amplifier to settle at small currents. One additional obstacle when measuring low currents is represented by leakage currents in the active feedback component, which can significantly skew the measured values of small currents [64].

On the other hand, linear transimpedance amplifiers have a smaller dynamic range (typically spanning 3–4 orders of magnitude), with their range being determined by the resistance value of the feedback resistor, which sets the current sensitivity. While conducting AFM experiments, the sensitivity can often be tuned manually or electronically, allowing the user to pick the current range which is most suitable for their experiment. However, this also requires prior knowledge on the expected current levels, which can be challenging due to variations across samples, probe geometries, and applied voltages. Despite this limitation, the bandwidth of TIAs is constant over the whole input range, allowing for a fast response time even at fA current ranges. Figure S2 shows the typical trade‐offs of SSRM measurements acquired using a logarithmic amplifier and a transimpedance amplifier: while the SSRM amplifier struggles at low currents, the TIA is limited by the smaller dynamic range. In the context of advanced logic nodes, where the volumes of doped regions are extremely small and only doping concentrations above ∼10^18^ at cm^−3^ are relevant, the limited dynamic range of TIAs becomes less of a constraint, as the orders of magnitude of doping are limited to a few decades. In addition to TIAs and logarithmic amplifiers, alternative solutions such as transimpedance amplifiers with real‐time gain adjustment [64] or switchable logarithmic‐linear current amplifiers [64] have also been demonstrated as options that combine the linear response of TIAs without sacrificing dynamic range. Despite the lower bandwidth (which limits the AFM scanning speed), these amplifiers are also promising solutions for the SSRM characterization of emerging transistor structures.

### 3.4 Carrier Mapping in Scaled Nanosheet‐Based Transistors

**FIGURE 5:**
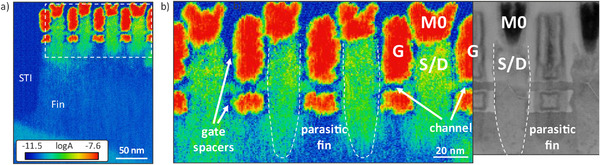
a) SSRM map of NSFET transistors. b) Enlarged detail corresponding to the dotted line in (a) and bright field HR‐TEM image of devices with the same structure. A direct comparison shows that carriers diffuse out of the S/D regions and reach the metal gate edges, entering the channel.

By incorporating optimal surface preparation, an adjusted force procedure with ultra‐sharp probes and a suitable current amplifier, it is possible to image NSFET transistors at dimensions relevant for cutting‐edge sub‐2nm nodes logic nodes. Figure 5a shows an SSRM map of sample A acquired using the PF‐TUNA linear amplifier with a current sensitivity of 2nA/V and sample bias (*V_Sample_*) of −0.5 V. A clear distinction can be made between the silicon semiconducting regions, the metallic gates, M0 contacts to S/D regions as well as the isolating layers in the STI and around the metals. By comparing the SSRM image with a bright field high‐resolution transmission electron microscopy (HR‐TEM) image of devices from the same wafer (Figure 5b), it is possible to observe the extent of electron diffusion from the highly doped Si:P S/D regions into the surrounding regions, as shown in the overlay image in Figure S3. In particular, carriers are observed to extend 3–7 nm laterally towards the inner‐spacers region in the nanosheet channel, up to the metallic gate edge. At the same time, this phenomenon is observed in the underlying silicon fin that constitutes the devices parasitic channel. In this case, the vertical extent of the diffusion is harder to estimate due to possible variations in the S/D cavity etch depth into the fin. Some variations are observed in carrier profiles in the channel of neighboring transistors: the absence of peculiar topography features that could affect the probe‐sample interaction and the repeatability of the observed features over subsequent measurements suggest that they could be related to doping inhomogeneities [65] rather than measurement artifacts. A clear indication that the observed contrast is related to the spreading resistance is given by the fact that by reducing the probe force by 17% the measured current in the S/D regions drops by ∼50% (Figure S4). This large decrease cannot be attributed solely to a reduction in contact radius alone, but rather to a limited Si‐I to Si‐II transformation, which increases sharply with small force increments in the low‐force regime [36]. As mentioned before, this highlights the need for careful force control to maintain an optimal balance between measurement stability and spatial resolution.

**FIGURE 6:**
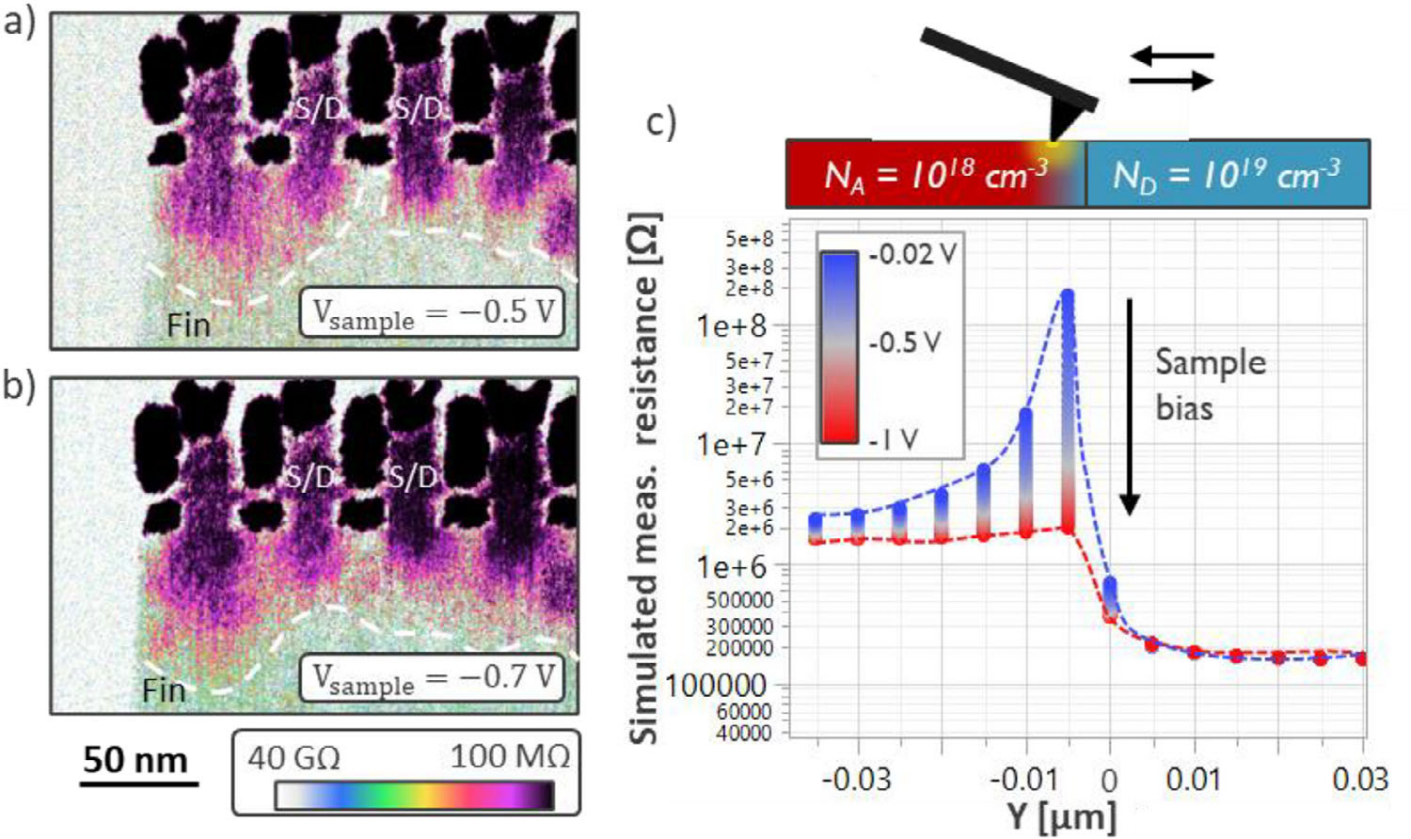
Measured spreading resistance while applying a sample bias of −0.5 V (a) and −0.7 V (b). c) Impact of sample bias on the TCAD simulated measured resistance while scanning over a p‐n junction (see Figure S5 for details). Increasing the sample bias masks the presence of the junction by lowering the measured resistance. Note that in the SSRM maps, the junction is not clearly visible due to the impact of surface states [49, 50].

In the same way, the sample bias has to be picked accordingly: while a large *V_Sample_* allows to overcome potential barriers at the probe‐sample contact, and therefore to increase the magnitude of measured currents and improve the S/N ratio, too large biases distort the spreading resistance measurements. This is visible in Figure 6, where the current acquired using the linear amplifier was used to compute the local spreading resistance at *V_Sample_* = −0.5 and −0.7 V (Figure 6a,b, respectively). In the latter image, it's visible how the measured resistance in the junction between the S/D regions and the ground‐plane doping in the sub‐fin is lower, and how the electrons from the S/D regions seem to spatially extend more downwards. To explain and confirm this effect, we simulated the measurement in a TCAD environment (Figure S5). The simulated structure of two portions of silicon in contact, one doped with 10^19^ cm^−3^ phosphorus atoms and the other with 10^18^ cm^−3^ boron atoms. The probe is represented by a half‐circular ohmic contact of 1 nm radius, while the back‐contact is placed 100 nm away. To simulate the impact of sample bias during SSRM scanning over junctions, multiple *I–V* characteristics were simulated while sweeping the back‐contact voltage and moving the grounded circular electrode in steps of 5 nm across the p‐n junction. Figure 6c shows that when the back contact is strongly biased, the resistance measured by the probe in proximity of the electrical junction is significantly lowered, until the junction is almost completely invisible to the measurement when *V_Sample_* = −1 V. This is due to the electric field‐induced band‐bending below the probe, which flattens the bands when scanning over the p‐n junction. When applying *V_Sample_* = +1 V, the effect is reversed, making the junction even sharper, promoting tunnelling, which also lowers the measured resistance (Figure S5b). The spatial extent of this effect is in the order of the Debye length, which is proportional to the inverse square root of the carrier concentration, making it particularly detrimental when imaging lowly doped areas and p‐n junctions. One might think that to avoid these undesired effects and measure the most realistic spreading resistance maps, the sample bias should be kept at a very small value. In practice, this is not always possible due to the Schottky nature of the SSRM contact. Current‐voltage spectroscopy measurements show that while the contact in the highly doped S/D regions is quasi‐metallic, in regions which are less doped, the contact is strongly rectifying (Figure S6). It is therefore necessary to apply a strong enough sample bias to overcome the contact barrier and measure sufficient current from the more resistive regions. Considering the results of the simulation in Figure 6, it was decided to fix *V_Sample_* to ‐0.5 V to ensure stable electrical contact while limiting electric field‐induced charge carrier displacements.

**FIGURE 7:**
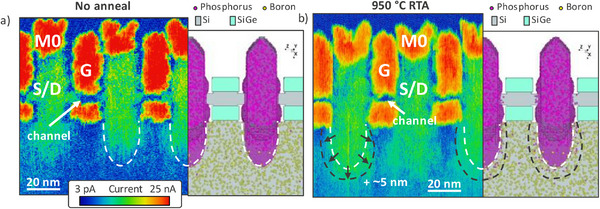
Comparison of SSRM with Kinetic Monte Carlo (KMC) phosphorus diffusion simulations for sample A (a) and B (b). Dashed lines surround high conductivity areas from S/D regions (white for sample A, black for sample B) and suggest ∼ 5 nm diffusion for sample B. A good agreement between simulations and SSRM suggests that the technique is capable of characterizing process steps in advanced device nodes.

Finally, we compare the impact of thermal budget on dopant diffusion and carrier distribution. A clear impact of the additional RTA is visible in the measured carrier distribution of sample A and sample B (Figure 7a,b, respectively). In particular, enhanced diffusion is observed in the devices sub‐Fin with a spatial extent of around 5 nm in the region of the S/D furthest away from the gate. This observation is consistent with known phosphorus diffusion mechanisms following annealing, which involve the release and migration of vacancy‐related complexes. These processes contribute to dopant profile broadening and redistribution in silicon [66]. The diffusion shape is also in very good agreement with semi‐atomistic Kinetic Monte Carlo (KMC) process simulations. The evolution of the outer boundary of the phosphorus atom cloud in the simulations after the 950 °C anneal clearly reproduces the experimentally observed SSRM profiles. In contrast, such a distinct difference in diffusion is not observed in the nanosheet channels. This may indicate carrier trapping due to traps at the interface between gate spacers and the nanosheet channel [11, 67] or even limited dopant activation and diffusion linked to enhanced vacancy injection from the nanosheet sidewalls [68, 69]. Although the latter phenomenon is simulated in KMC simulations, the parameters employed are only calibrated to micrometer‐large structures and might not be accurate for nanoscale volumes. However, the SSRM technique is also impacted when measuring in confined volumes. SSRM is quantitative only when the spreading resistance dominates, which may not hold true in nanosheet channels due to current confinement effects [70] In this context, Fast Fourier Transform SSRM (FFT‐SSRM) has been proposed as a method to isolate the spreading resistance signal and address this limitation [52, 71], which will be explored in future works. At the same time, the tip‐induced phase transformation is also inhibited in confined volumes [70, 72, 73]. This suppression complicates the interpretation of the data, as the associated increase in measured resistance becomes convoluted with true variations in the carrier distribution maps. Despite these quantitative limitations in confined volumes, SSRM remains highly valuable for process split comparisons, offering spatially resolved information on carrier distributions that complements both device simulations and electrical characterization.

### 3.5 Considerations on Reproducibility and Metrology Applications

To become a consolidated metrology technique, SSRM must provide high‑resolution, high‑fidelity data and operate reproducibly across samples and tools. This depends on several practical factors. A first requirement is a stable sample‑preparation sequence: surface quality and back‑contact integrity directly influence the spreading‑resistance signal, making consistent polishing and reliable back‑contact formation essential for routine sub‑nanometer topography and stable electrical access. A second factor is probe‑to‑probe variability. Commercial diamond probes exhibit apex radii in the 5–15 nm range, which strongly affects the contact area, the achievable spatial resolution, and the force required to reach the Si‑II phase transformation. Reducing this variability through tighter manufacturing tolerances or improved apex characterization would significantly enhance reproducibility and reduce the fine‑tuning needed for each sample. Quantitative accuracy in confined device volumes remains a limitation. The scaled geometry, contacting scheme, and strong current‑confinement effects prevent the use of conventional calibration structures typically employed for absolute carrier‑concentration extraction in planar structures. Approaches such as FFT‑SSRM or device‑specific calibration strategies that explicitly account for confined volumes will be needed to enable metrology‑grade quantification. Finally, SSRM's metrology potential may be extended through less‑invasive access strategies. Demonstrations of SSRM performed directly on TEM lamellae [39] show that localized, non‑destructive cross‑section analysis is feasible without consuming an entire wafer, opening possibilities for future inline or near‑inline implementations.

## 4 Conclusion

In this work, we successfully overcome the spatial resolution limits of SSRM in carrier mapping within complex 3D architectures. We demonstrate its capability in sub‐2 nm node NSFETs with ultra‐thin channels as thin as 5.5 nm. This is achieved by a holistic methodology combining optimized cross‐section preparation, sharp diamond probes and ultralow forces that minimize the electrical contact radius, and a linear current amplifier to accurately capture the large, rapid resistance variations in these complex structures. We directly compare the impact of an additional RTA step on carrier distribution, showing a distinct enhancement of phosphorus diffusion, extending approximately 5 nm after annealing. This experimental finding shows excellent qualitative agreement with semi‐atomistic KMC process simulations, validating both the measurement technique and the process model. Despite the remaining challenges in achieving fully quantitative interpretation within the nanosheet itself, this study unequivocally establishes the new SSRM methodology as a powerful tool for process split comparisons. It provides spatially resolved, direct information on carrier placement that is complementary to electrical characterization and vital for the optimization of junction engineering in the most advanced GAA transistor architectures. The developments presented here also point toward the broader potential of SSRM for metrology applications to support device scaling.

## Funding

A.P. acknowledges Research Foundation – Flanders (FWO) for the Strategic Basic Research PhD fellowship grant 1S20225N. This work has been enabled in part by the NanoIC pilot line. The acquisition and operation are jointly funded by the Chips Joint Undertaking, through the European Union's Digital Europe (101183266) and Horizon Europe programs (101183277), as well as by the participating states Belgium (Flanders), France, Germany, Finland, Ireland and Romania.

## Conflicts of Interest

The authors declare no conflicts of interest.

## Supporting information




**Supporting File**: smtd70550‐sup‐0001‐SuppMat.docx.

## Data Availability

The data that support the findings of this study are available from the corresponding author upon reasonable request.
