# Supplementary material for: Carrier Mapping in Sub‐2nm Node Nanosheet Transistors with Scanning Spreading Resistance Microscopy
Source: Small Methods. 2026 Feb 10;10(5):e02279. doi: 10.1002/smtd.202502279 (PMC12972251; doi:10.1002/smtd.202502279)

Supporting Information

**Carrier Mapping in sub-2nm Node Nanosheet Transistors with Scanning Spreading Resistance Microscopy**

Andrea Pondini*, Pierre Eyben, Lennaert Wouters, Albert Minj, Thomas Hantschel, Philippe Matagne, Jérôme Mitard, Anne Verhulst

A. Pondini, P. Eyben, L. Wouters, A. Minj, T. Hantschel, P. Matagne, J. Mitard, A. Verhulst

imec vzw, Leuven, Belgium

E-mail: andrea.pondini@imec.be

A. Pondini, A. Verhulst

Dept. of Electrical Engineering (ESAT), KU Leuven, Leuven, Belgium

**Figure S1.** A dedicated SSRM test structure was designed, featuring groups of transistors processed next to each other over an area of 15×20 μm. These dimensions are needed to ensure that the module can be reliably targeted during cross-section preparation. The devices span over rows with a pitch of 51 nm (fin pitch), with shared M0 contacts and gate lines running perpendicular to the nanosheet channel direction. The M0 lines are connected to M1 and then routed to a standard metal pad, with the gate lines following the same connection pattern. Wafer dies were cleaved down into small coupons and a cross-section along the [110] channel direction was prepared for SSRM measurements. In each coupon, trenches were milled on both sides of the SSRM module from the top surface using a focused-ion-beam (FIB) to easily locate the region of interest from the cross section. Then, an electrical back contact was prepared by FIB milling a trench through the SSRM module’s S/D contact pad, followed by localized platinum deposition within the trench. Additionally, a 20 nm Pt layer was deposited over the entire top surface to facilitate back-contacting the pad. After mechanically scratching the sample, silver paste was applied to establish electrical contact with both the substrate and the topmost Pt layer.


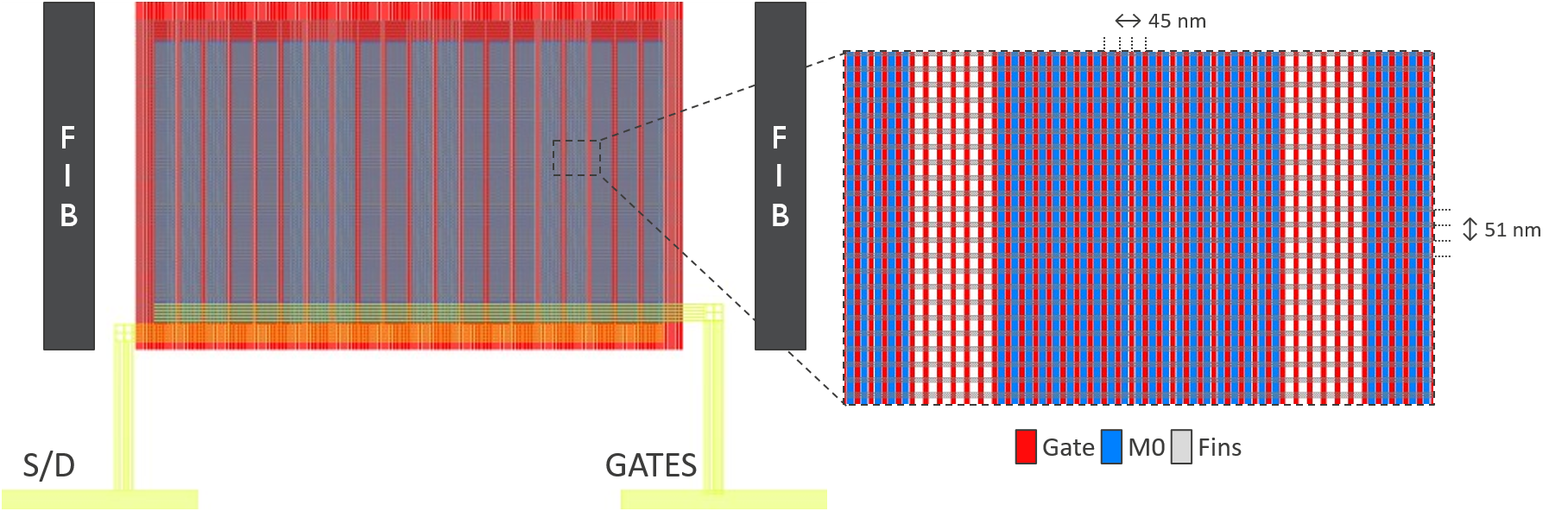


**Figure S2.** SSRM measurements of silicon sample doped with different concentrations of boron, acquired using a cone probe and employing a logarithmic SSRM amplifier (a) and a transimpedance amplifier (PF-TUNA), respectively. The sample bias was varied during the scans, sectioning the SSRM data in multiple rows. While the SSRM amplifier is uncapable of measuring small currents (see measurements on lowly doped regions measured with small positive bias), the linear amplifier has limited dynamic range and can image only a few doping layers for each sample bias.


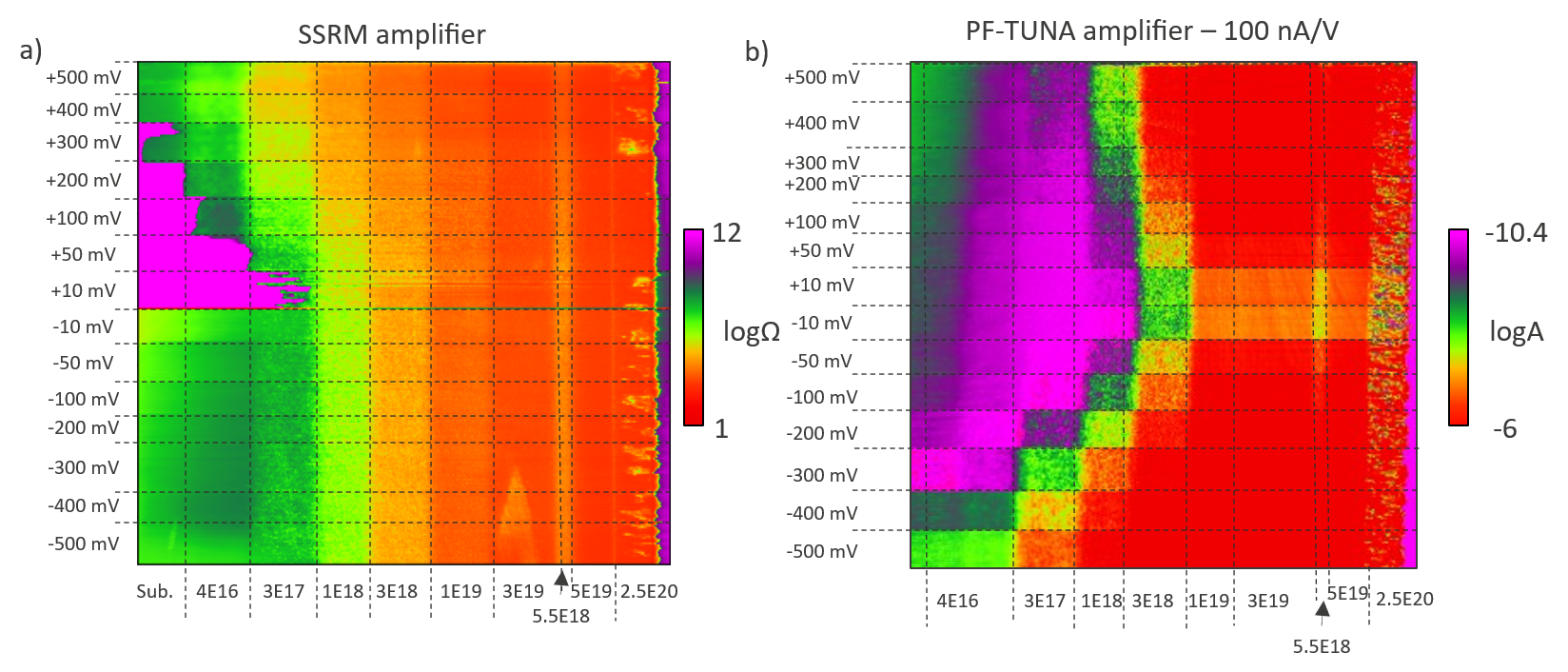


**Figure S3.** SSRM data (center) is used as color information and overlayed with a 25% opacity (right) on a HR-TEM image (left) of devices of the same dimensions.
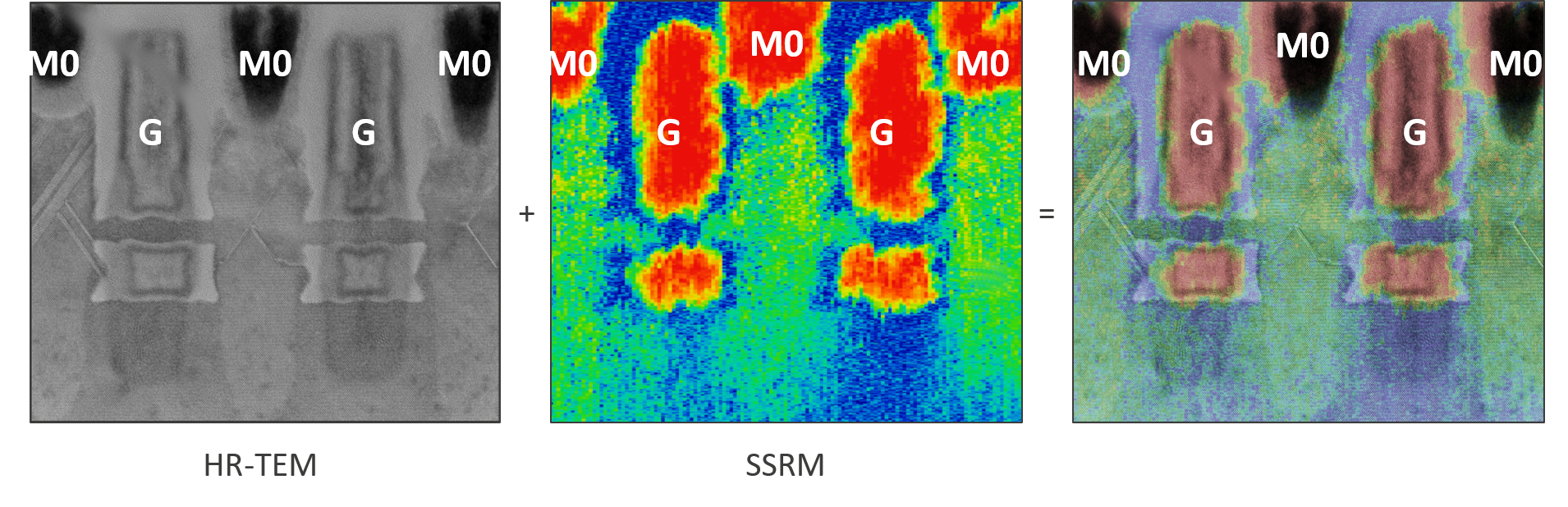


**Figure S4.** Current channel obtained during SSRM at two different AFM deflection setpoints, resulting in two different applied forces. When lowering the force, the SSRM contrast between the source/drain regions and the surrounding silicon is degraded, suggesting that the silicon phase transformation needed for SSRM is limited.
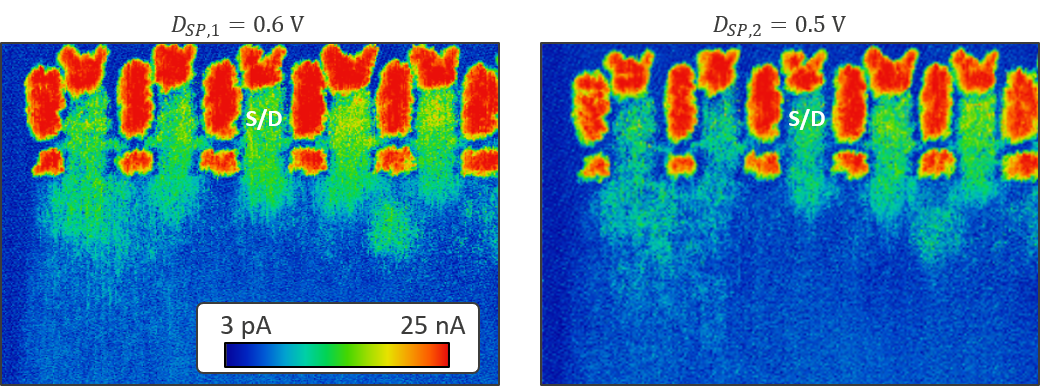


**Figure S5.** a) TCAD simulation set-up: a 1 nm ohmic circular electrode represents the probe and is moved across a p-n junction while the voltage of a back-contact is swept and the current is determined (see Figure 6c). b) Band diagrams near the surface showing the effect of tip-induced band-bending at different back-contact voltages.


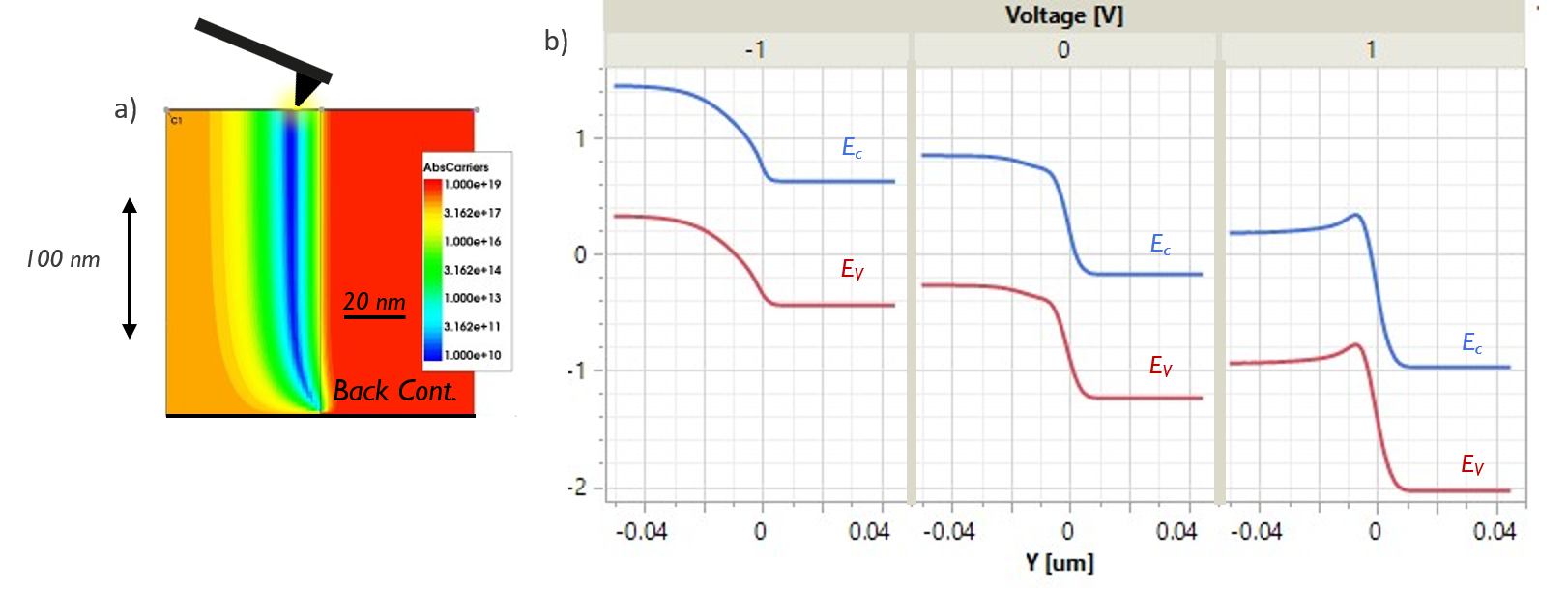


**Figure S6.** a) SSRM map and corresponding I-V spectroscopy data (b) acquired on position 1 and 2.


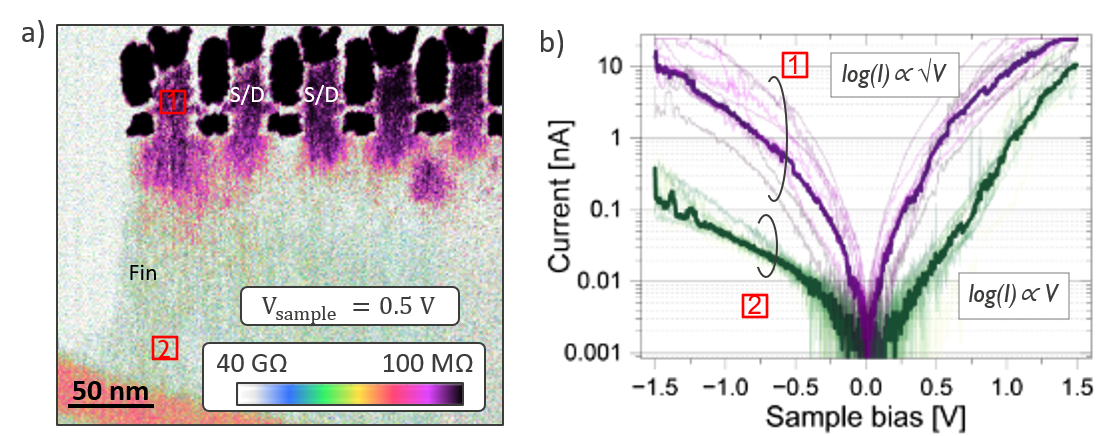

Supplement: Supplementary file 1 — Supporting File: smtd70550‐sup‐0001‐SuppMat.docx. [file SMTD-10-e02279-s001.docx]
